# Prevalence of attention deficit hyperactivity disorder in homeless children and adolescents: A systematic review and meta-analysis

**DOI:** 10.1080/19585969.2025.2486355

**Published:** 2025-04-02

**Authors:** Charles Denis, Denis Boucaud-Maitre, Jérôme Brunelin, Lucie Jurek, William Vallet, Caroline Demily

**Affiliations:** a Centre Hospitalier le Vinatier, Bron, France; b Equipe EPICLIV, Université des Antilles, Fort-de-France, Martinique; c Université Claude Bernard Lyon 1, CNRS, INSERM, Centre de Recherche en Neurosciences de Lyon CRNL U1028 UMR5292, PSYR2, Bron, France; d Université Claude Bernard Lyon 1, RESHAPE Inserm U1290, Lyon, France; e Centre de Référence Maladies Rares Troubles du Comportement d’Origine Génétique (GénoPsy Lyon), Centre d’excellence Autisme iMIND, Le Vinatier Etablissement Lyonnais référent en psychiatrie et santé mentale, UMR 5229, CNRS & Université Lyon 1, Lyon, France

**Keywords:** Homeless, children and adolescents, ADHD, prevalence

## Abstract

**Introduction:**

This systematic review and meta-analysis aimed to examine the prevalence of Attention deficit hyperactivity disorder (ADHD) in homeless children and adolescents, and the factors that may influence its prevalence.

**Methods:**

Relevant publications in Medline, Web of Science, Scopus and PsycINFO were systematically searched to identify studies on the prevalence of ADHD in homeless children and adolescents (≤19 years). The extracted data were pooled using a random-effects model.

**Results:**

Thirteen studies involving 2878 homeless children and adolescents were included (mean age: 12.0 years, sex F/M: 0.43). The prevalence rates of ADHD vary considerably across studies, ranging from 1.6% to 64.5%. The pooled prevalence of ADHD was 22.8% (95% CI 12.9–34.4%, *I*^2^ =98%). Meta-regression analyses indicated that age (slope = 0.046; *p* = .042) significantly increased ADHD prevalence. The prevalence of ADHD in studies with a mean age ≥ 12 years (43.1%, 95% CI 26.5–60.4%) was higher than those with a mean age < 12 years (13.1%, 95%CI 4.3–25.6).

**Conclusion:**

Despite the high heterogeneity of the studies, we observed that ADHD could affect almost a quarter of homeless children and adolescents. Reintegrating them into care systems and ensuring access to public health interventions tailored for homeless families and youth is imperative for breaking the cycle of homelessness and improving long-term trajectories.

## Introduction

According to the United Nations, 150 million children live on streets worldwide, and this major concern is not limited to low- and middle-income countries (The Lancet Child Adolescent Health 2023). In the United States, approximately 1.5 million children experience homeless annually (Grattan et al. 2022). In Europe, it is difficult to provide an overall number of homeless people among youths and children, but some countries have reported a large presence of children and youth among homeless people. In Ireland, they represent 38% of the homeless population and 17% in Bosnia (Baptista and Marlier 2019). In England, approximately 210,000 homeless children live in temporary accommodations (Rosenthal et al. 2020). Definitions of children, adolescents, and even young people who are homeless may vary slightly among health advocates, government agencies (The Lancet Child Adolescent Health 2023), and clinical researchers, but it is often agreed that these are individuals who do not have a fixed, regular, and adequate night-time residence. There are some distinctions between homelessness trajectories (temporary disconnected, unstably disconnected or chronically disconnected), between street-living, street working and street family (United Nations 2012) or between street and shelter users.

Homeless children and adolescents remain largely invisible, and few studies have focused on their health and well-being (García Murillo et al. 2016). Homelessness is associated with a greater risk of health problems (Morton et al. 2018), violence (Heerde et al. 2014), early pregnancy (Greene and Ringwalt 1998), substance use (Embleton et al. 2013), vaccine-preventable infectious disease (Gultekin et al. 2020), suicidal behaviour, and early mortality (Auerswald et al. 2016). Given their precarious and difficult living conditions, homeless children and adolescents are also at a greater risk of mental disorders. The prevalence of mental health problems among homeless school-age children is two to four times higher than that among housed children (Bassuk et al. 2015; Vandentorren et al. 2016). The rates of anxiety, conduct disorders, post-traumatic stress disorder, and major depression are higher and mental health problems are often diagnosed late or incompletely (European Commission, 2021).

Surprisingly, Attention-Deficit/Hyperactivity Disorder Childhood (ADHD) has received little attention. ADHD is a developmental disorder associated with an ongoing pattern of inattention, hyperactivity, and/or impulsivity. Its prevalence in children and adolescents varies between 3% and 7.6% among studies (Sayal et al. 2018; Chaulagain et al. 2023; Salari et al. 2023), with a global consensus of around 5.9% (Faraone et al. 2021), and is two–three times higher in boys. Comorbidities are commonly observed in ADHD, including other neurodevelopmental disorders, learning disorders, externalising and internalising disorders (Gnanavel et al. 2019) or somatic conditions, in particular dermatitis, rhinitis or obesity (Arrondo et al. 2022).

Moreover, ADHD present similar unfavourable heaths events than homeless youth, notably drug and alcohol addiction, injuries, depression, psychotic disorders, suicidal spectrum behaviour and criminality (Taurines et al. 2010; Garas and Balazs 2020; Nourredine et al. 2021; Chaulagain et al. 2023). There is a close relationship between childhood ADHD and homeless. Using a 33-years cohort study, a significant risk of homelessness (OR = 3.60) was observed among males with childhood ADHD (García Murillo et al. 2016). However, in the literature (Hossain et al. 2020), only two reviews (Hodgson et al. 2013; Bassuk et al. 2015) have focused on the prevalence of mental disorders in homeless youths, among which ADHD was an outcome. Hodgson et al. (Hodgson et al. 2013) identified only two studies and Bassuk et al. (Bassuk et al. 2015) identified only one study.

It is necessary to investigate the prevalence of ADHD in homeless children and adolescents to inform clinicians and policymakers, leading to potential specific interventions and healthcare services they need. Indeed, homeless children and adolescents suffer from poor access to healthcare and services, whereas ADHD is mostly treatable with pharmacological treatments or behavioural interventions. Thus, we conducted a systematic review and meta-analysis of the prevalence of ADHD among homeless children and adolescents (aged ≤19 years).

## Methods

The study protocol was registered in PROSPERO (CRD42024534078). This systematic review adheres to the Preferred Reporting Items for Systematic Reviews and Meta-Analyses (PRISMA) statement (Page et al. 2021).

### Search strategy

The literature search was conducted on December 14, 2023 using the following scientific databases: MEDLINE, PsycINFO, SCOPUS and WEB OF SCIENCES. The search strategy included a set of terms incorporing alternative descriptors, specifically: ‘Homeless’ AND ‘Attention Deficit Disorder’, with no age restriction. This strategy, developed and executed by a scientific librarian, is detailed for each database in Appendix 1. Studies were included irrespective of the language of publication. Additionally, the reference lists of the retrieved articles were reviewed, and potentially relevant papers that met the inclusion criteria were identified and retrieved. There were no limitations on the type of articles considered; original studies, letters to the editor, and abstracts from meetings and conferences were included.

### Eligibility criteria and study selection

The inclusion criteria were as follows: participants had to be homeless; ADHD had to be screened or diagnosed using a scale, self-report, or clinical examination; participants had to be under 19 years of age at baseline, corresponding to the definition of children and adolescents (Sawyer et al. 2018); and studies had to report both the number of participants and the number of ADHD cases. The exclusion criteria were as follows: studies that did not contain empirical data or those sampled from selected subpopulations such as inpatients.

Study selection was a two-stage process. First, the titles and abstracts identified in the initial search were screened by two authors (C.D. and D.B.M.). Second, the full-text reports of potentially relevant studies were assessed by the same reviewers, and only studies pertaining to homeless children and adolescents were selected. In cases of disagreement, the articles were carefully re-evaluated by both authors, and the final selection was based on their consensus.

### Data collection and quality assessment

Data extraction was completed independently by the two authors (C.D. and D.BM.). Information extracted from the studies included authors, year, study setting or location, study design, sample size, homeless definition, recruitment source, age and gender of participants, substance use, diagnostic tool used and prevalence data.

To assess the methodological quality of the selected studies, we used the Joanna Briggs Institute (JBI) Critical Appraisal Checklist for Studies Reporting Prevalence Data (Migliavaca et al. 2020; Munn et al. 2020). The tool consists of nine items: (Q1) Was the sample frame appropriate to address the target population? (Q2) Were study participants recruited in an appropriate way? (Q3) Was the sample size adequate? (Q4) Were the study subjects and setting described in detail? (Q5) Was data analysis conducted with sufficient coverage of the identified sample? (Q6) Were valid methods used for the identification of the condition? (Q7) Was the condition measured in a standard, reliable way for all participants? (Q8) Was there appropriate statistical analysis? (Q9) Was the response rate adequate, and if not, was the low response rate managed appropriately?

The assessment was performed by two authors (C.D. and D. B. M.), using a four-choice response scale (yes, no, unclear, and not applicable), and any differences were resolved through discussion. Specifically, for Q1, we examined whether homelessness was defined and the percentage of male included. For Q3, a sample size inferior to 50 children and/or adolescents was considered insufficient for a prevalence study. For Q4, we scrutinised the recruitment source, particularly focusing on whether participants were rough sleeping, living in a shelter, or in temporary accommodation. For Q6 and Q7, a ‘no’ response indicated that the study used an imprecise scale. Studies with an overall quality assessment score of more than 50% (i.e., five out of nine) ‘yes’ responses were classified as moderate to high quality.

### Statistical analysis

Quantitative analyses were conducted using the R project with the meta package (Balduzzi et al. 2019), the metafor package (Viechtbauer 2010) and the dmetar packages (Harrer et al. 2019). The prevalence and 95% confidence intervals (CI) of ADHD were calculated using a random-effects model and the Freeman Tukey double arcsine transformation (Freeman and Tukey 1950). Heterogeneity between studies was measured by *τ*^2^ and *I*^2^ statistics, with *I*^2^> 50% indicated high heterogeneity (Higgins et al. 2003). Finally, Egger’s test (weighted regression models with multiplicative dispersion) was employed to test funnel plot asymmetry and potential publication bias (Higgins et al. 2003).

To investigate the effect of continuous moderators (age, quality score, years of publication, sex ratio, and sample size) on the prevalence of psychiatric diseases, a meta-regression was performed using a mixed-effects model.

Subgroup analyses corresponding to categorical moderator analysis were conducted using a mixed-effects model based on the summary of effect size for each subgroup derived from the random-effects model. Subgroup analyses were conducted based on economic group (low or middle income/high income countries), mean age (<12 years versus ≥ 12 years; the mean age was calculated when only categories were available), location (USA versus other countries) and the type of ADHD assessment tools used (screening tools such as SDQ, ADI, CBCL, and Vanderbilt scales versus diagnostic tools such as DISC-IV, MINI-KID, medical records, and psychiatrist diagnosis). Potential moderating effects on the prevalence of ADHD included sample size, mean age, proportion of females, publication year, and quality assessment score. Funnel plots and Egger’s regression model (Egger et al. 1997) were used to test for publication bias.

## Results

### Study characteristics and quality assessment

**Figure 1.**
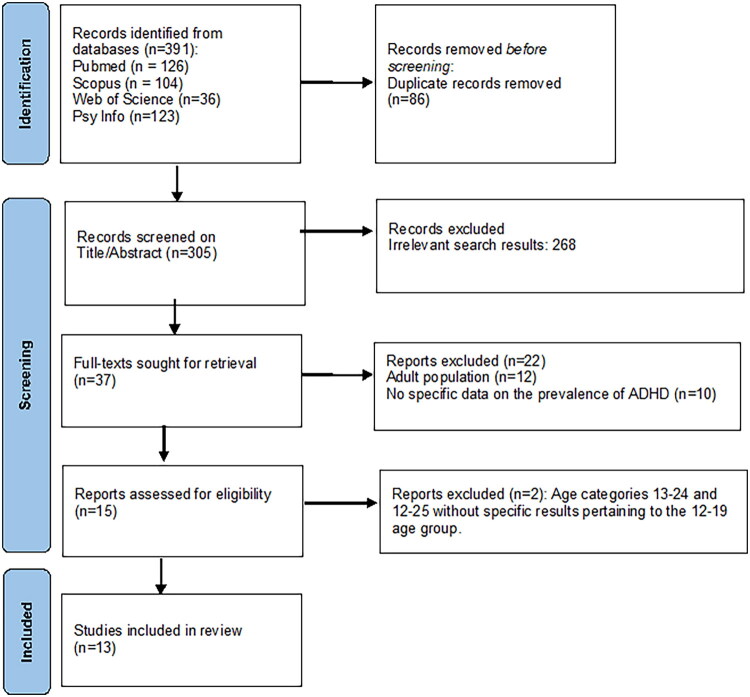
Flowchart of the selection of studies.

The initial search strategy identified 391 studies. After removing 86 duplicates, 305 studies were screened on the basis of their titles and abstracts and 37 full-texts were sought for retrieval. The full-text screening of these articles eliminated 22 articles that still did not meet the inclusion criteria. They concerned only studies carried out in adults or without specific results on the prevalence of ADHD (see appendix 1). Ultimately, 15 studies met the inclusion criteria (Figure 1). Two eligible studies were excluded due to participant ages between 13 and 24 years (mean age: 20 years) (Narendorf et al. 2017) or 12–25 years (mean age: 19.6 years) (Middleton et al. 2018) without specific results for the 12–19 age range. The study by Zemanek et al. (Zemanek et al. 2020), which included children and youths aged 11–20 years with a mean participant age of 15.3 years, was not excluded. Thus, this systematic review ultimately included 13 studies (*n* = 2878) (Unger et al. 1997; Cauce et al. 2000; Grant et al. 2007; Yu et al. 2008; Silva et al. 2010; Hayes et al. 2013; Ojha et al. 2013; Taib and Ahmad 2014; Oppong Asante et al. 2015; Lewis and Lewis 2016; Roze et al. 2016; Labelle et al. 2020; Zemanek et al. 2020), comprising 10 original articles, 2 meeting abstracts (Lewis and Lewis 2016; Zemanek et al. 2020), and one letter to the editor (Silva et al. 2010).

Among the 13 studies included, 11 reported the mean age of the participants, with an average 12.0 years old. Based on data from 11 studies, the proportion of males was 56.8%. One study conducted in Iraq (Taib and Ahmad 2014) focused exclusively on homeless boys. Geographically, seven studies were conducted in the USA (*n* = 1578) (Unger et al. 1997; Cauce et al. 2000; Grant et al. 2007; Yu et al. 2008; Hayes et al. 2013; Lewis and Lewis 2016; Zemanek et al. 2020), while the remaining six studies included homeless children and adolescents from various countries, including Canada, Ghana, Iraq, France, Brazil, and Nepal (Silva et al. 2010; Ojha et al. 2013; Taib and Ahmad 2014; Oppong Asante et al. 2015; Roze et al. 2016; Labelle et al. 2020). In seven studies, participants were accommodated in shelters, hostels, or transitional living facilities (Yu et al. 2008; 2008; Hayes et al. 2013; Ojha et al. 2013; Lewis and Lewis 2016; Labelle et al. 2020; Zemanek et al. 2020), whereas participants were rough sleeping in two studies (Taib and Ahmad 2014; Oppong Asante et al. 2015). Two studies involved mixed samples (Unger et al. 1997; Cauce et al. 2000).

Four studies explicitly defined homelessness as part of their inclusion criteria (Unger et al. 1997; Cauce et al. 2000; Silva et al. 2010; Roze et al. 2016). Studies with mean participant ages less than or equal to 12 years primarily focused on homeless families, whereas those with participants over 12 years old primarily involved isolated homeless individuals. Only four studies reported the use of illicit substances (Cauce et al. 2000; Silva et al. 2010; Lewis and Lewis 2016; Zemanek et al. 2020). Three studies indicated an overall substance use prevalence of 40%–60%, with alcohol consumption ranging between 10% and 30%. One American study reported the use of hard drugs among 10% of adolescents (Cauce et al. 2000).

ADHD screening methods varied across the included studies. The Diagnostic Interview Schedule for Children (DISC-IV) was utilised in three studies (Cauce et al. 2000; Yu et al. 2008; Labelle et al. 2020), while the Strengths and Difficulties Questionnaire (SDQ) was employed in three other studies (Hayes et al. 2013; Oppong Asante et al. 2015; Roze et al. 2016). Additionally, two studies conducted screening through the extraction of medical documentations (Grant et al. 2007; Zemanek et al. 2020).

For the remaining studies, alternative tools were used: the Adolescent Diagnostic Interview (ADI) (Unger et al. 1997), Mini International Neuropsychiatric Interview for Children and Adolescents (MINI-KID) structured interviews (Taib and Ahmad 2014), Child Behaviour Checklist (CBCL) (Ojha et al. 2013), and Vanderbilt Assessment Scale-Parent Report (VADPRS) (Lewis and Lewis 2016). In one study, diagnosis of hyperkinetic disorder was performed by a trained psychiatrist (Silva et al. 2010) (Table 1).

**Table 1.** Characteristics of the studies included in the meta-analysis.

Study (Year), country	Homeless definition used	Recrutment source	With or without family	Age category	Age	Gender (%men)	Substance use	Diagnosis or screening tool	Sample size	Number of cases	Prevalence (95% CI)
Labelle et al. (2020), Canada (Labelle et al. 2020)	Not defined	Temporary shelters	Without family	12–19	12–15: 55.3% 16–19: 44.7% Mean age*: 15.3	43%	Not reported	DISC-IV	76	49	64.5% (53.7–75.2)
Oppong Asante et al. (2015), Ghana	Not defined	Street children	Not precised	8–19	12.6	53.7%	Not reported	SDQ	227	122	53.9% (47.3–60.2)
Unger et al. (1997), USA	Primary night residence is in a supervised public or private shelter, an institution or a publico r private place not ordinarily used (street, park, abandoned car or building)	Street children or shelters	Without family	12–18	13–15: 15.9% 16–18: 84.0% Mean age*: 17.0	64.3%	Not reported for 12–18	ADI (Adolescent Diagnostic interview)	188	42	22.1% (16.4–29.6)
Taib and Ahmad (2014), Irak	Not defined	Street children	Homeless family	8–16	8–12: 58% 13–16: 42% Mean age*: 11.9	100%	Not reported	MINI	100	5	5.0% (0.7–9.3)
Ojha et al. (2013), Nepal	Not defined	Shelters	Without family	6–18	6–10: 27% 11–15: 61.1% 16–18: 11.9% Mean age*: 11.3	45.2%	Not reported	CBCL/6–18	122	2	1.6% (0.0–3.9)
Silva et al. (2010), Brazil	Children and adolescents that are separated from their amily because they had run away from home or were sent to foster centres by the Justice System and, sometimes, their siblings whoare still at home.	Imprecise (referred to The Equilibrium Project)	Without family	12–19	Not reported	68%	40.4%	Psychiatric diagnosis	351	57	16.2% (12.4-20.1)
Roze et al. (2016), France	A person is considered to be homeless on any given day if he or she spent the previous night in a sheltered accommodation or slept in a place not intended for living	Housing facilities, Emergency shelters	Homeless family	4–13	4–6: 34.5% 6–13: 65.5% Mean age*: 7.6	47.2%	Not reported	Psychologist, SDQ	424	10	2.4% (0.9–3.8)
Cauce et al. (2000), USA	No viable residence (e.g., on the streets or in emergency shelters) or stable residence (e.g., staying with friends on a temporary basis), who were not in the custody of the State.	Street children or shelters	Without family	13–17	13–15: 29.4% 16–17: 43.1% 18–21:27.5% Mean age: 16.4	58%	Alcohol: 35% Cannabis: 27% Hard drugs: 10%	DISC-R	261 13–15: 105 16–17: 156	89 34 55	34.1% 32% 35%
Zemanek et al. (2020), USA	Not defined	Homeless adolescent shelter	Without family	11–20	15.3	42.5%	Illicite drug: 47.1% Alcohol: 12%	Medical file	239	104	43.5%
Lewis and Lewis (2016), USA	Not defined	Local City Mission	Homeless family	school-aged children	10.8	79.1%	Drug use: 59.5% Alcohol: 13%	Parent reported Parent Vanderbilt Assessment Scale	115	37	32%
Hayes et al. (2013), USA	Not defined	Emergency shelters, transitional housing, permanent supportive housing	Homeless family	3–11	Not reported	Not reported	Not reported	SDQ	264	90	18%
Yu et al. (2008), USA	Not defined	Shelter locations	Homeless family	5–16	Not reported	Not reported	Not reported	DISC	202	12	6%
Grant et al. (2007), USA	Not defined	Shelters or in clinics established on-site at shelters.	Homeless family	3–19	3–4 years: 26.2% 5–11: 50.8% 12–19: 23% Mean age*: 8.5	45%	Not reported	Electronic health Records	309	64	20.7%

*Mean age based on age categories group, 95% CI: confidence interval.

### Risk of bias

The median quality score was six out of nine (see Appendix 2). Among the studies included in the meta-analysis, three studies were deemed to have a high risk of bias (Yu et al. 2008; Lewis and Lewis 2016; Zemanek et al. 2020). This assessment was based on several factors, including inadequate reporting of relevant information on study subjects and setting, lack of reporting on mean age and gender (Yu et al. 2008), or utilisation of uncertain tools for diagnosing ADHD (Lewis and Lewis 2016). Overall, only five studies reported participation rates.

### Prevalence of ADHD

**Figure 2.**
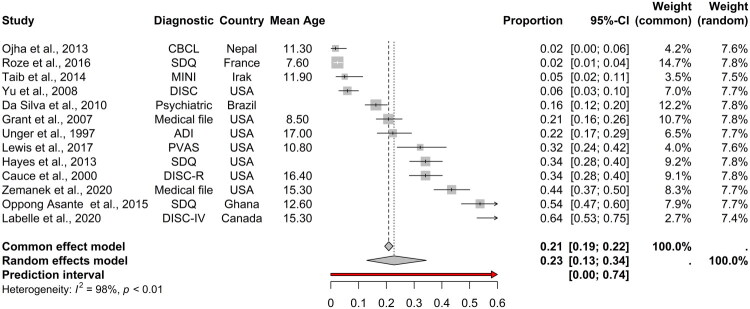
Forest plot of the prevalence of ADHD among children and youths homeless.

**Figure 3.**
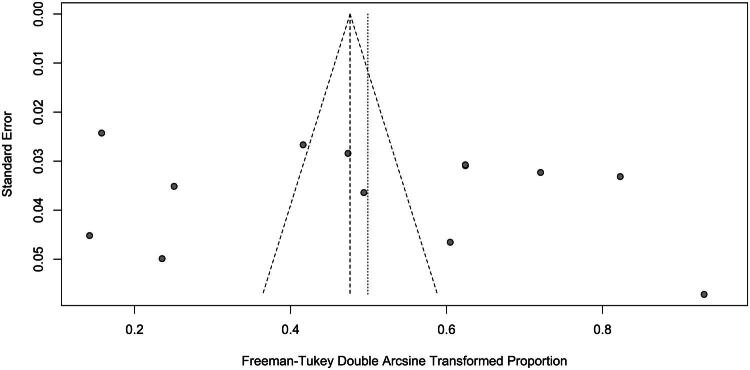
Funnel plot of the 13 included studies reporting the prevalence of ADHD among children and youths homeless.

The prevalence rates of ADHD among homeless children and adolescents vary considerably across studies, ranging from 1.6% to 64.5%. The studies reporting the lowest prevalence rates were conducted in Nepal (1.6%) (Ojha et al. 2013), France (2.4%) (Roze et al. 2016) and Iraq (5%) (Taib and Ahmad 2014), whereas the highest prevalence rate was reported in Canada (64.5%) (Labelle et al. 2020). The pooled prevalence of ADHD among homeless children and adolescents, derived from the meta-analysis, was 22.8% (95% CI 12.9%–34.4%, *τ*^2^ = 579.9, *I*^2^ = 98%, *p* < .001) (Figure 2). Notably, no significant effect was detected by Egger’s test, indicating a symmetrical forest plot and no significant evidence of potential publication bias (*t* = 0.80, *p* = .440) (Figure 3).

The results of the meta-regression analyses indicated that older age was associated with a statistically significant increase in the prevalence of ADHD (slope = 0.046; *p* = .042). Other factors, such as the proportion of males (slope = −0.376; *p* = .403), quality score (slope = −0.0176; *p* = .559), year of publication (slope = 0.007; *p* = .470), and sample size (slope = −0.005; *p* = .409), did not significantly moderate the prevalence of ADHD among homeless children and adolescents.

Subgroup analyses, as shown in Table 2, revealed that the prevalence of ADHD in studies with a mean age ≥ 12 years (43.1%, 95% CI 26.5%−60.4%) was significantly higher than in those with a mean age < 12 years (13.1%, 95% CI 4.3%−25.6%) (*Q* = 8.17, *p* = .004). No significant differences were observed between (i) studies conducted in the United States compared to other countries, (ii) low- and middle-income countries (Ghana, Iraq, Brazil, and Nepal) compared to high-income countries (USA, Canada, and France), or (iii) screening compared to diagnosis ADHD assessment tools.

**Table 2.** Subgroup analyses of the prevalence of ADHD among children and youths homeless.

Subgroups	Categories (number of studies)	Prevalence (%)	CI 95%	Events	Sample Size	τ^2^ (*p*)	*I* ^2^	*p*	Q (*p* value between subgroups)
Mean age	Age < 12 (*n* = 6)	13.1%	4.3–25.6	208	1334	0.038 (<.001)	97.7%	.004	8.17
	Age ≥ 12 (*n* = 5)	43.1%	26.5–60.4	406	991		94.1%		
Location	USA (*n* = 7)	26.5%	12.8–43.0	438	1578	0.054 (*p* < .001)	95.1%	.477	0.50
	Other countries (*n* = 6)	18.6%	6.3–35.3	245	1300		98.7%		
Income	Low or middle incomes countries (*n* = 4)	15.4%	2.3–36.5	186	800	0.054 (<.001)	98.4%	.368	0.81
	High incomes countries (*n* = 9)	26.3%	13.4–41.7	497	2078		97.9%		
ADHD assessment tool	Screening tools (*n* = 6)	22.5	9.2–39.5	303	1340	0.054 (*p* < .001)	98.5%	.959	0.00
	Diagnostic tools (*n* = 7)	23.1%	8.7–41.7	380	1538		97.4%		

## Discussion

This systematic review and meta-analysis represents the first comprehensive assessment of ADHD prevalence in homeless children and adolescents. Despite the high heterogeneity of prevalence results from the 13 studies, we determined that the prevalence of ADHD in this population was 22.8% (95% CI: 12.9–39.4). This result contrasts with the 5.9% prevalence reported in non-homeless children and adolescents, as per the World Federation of ADHD International Consensus Statement (Faraone et al. 2021). Our findings align with a growing body of literature that highlights the heightened prevalence of developmental and psychiatric disorders among homeless children and adolescents. The observed association between homelessness and ADHD suggests a particular susceptibility to housing insecurity within this vulnerable population. Although our study did not explore the familial backgrounds of the children and adolescents studied, it is plausible that a significant hereditary component may contribute to ADHD susceptibility, as heritability estimates for ADHD typically range from 74% to 80% (Chen et al. 2017; Faraone et al. 2021). Additionally, potential gene-environment interactions may contribute to the development of ADHD symptoms (Tsai et al. 2020; Einziger and Berger 2022; Schwabe et al. 2024). Certain individuals may be more sensitive to their home and family environments, thereby increasing their susceptibility to ADHD symptoms (Martel et al. 2011; Auerbach et al. 2017).

The different instruments used to screen for or diagnose ADHD may account for the high observed heterogeneity in prevalence. Notably, critical information for the diagnosis of ADHD was often missing and half of the studies used only screening scales. ADHD should be diagnosed by a licenced clinician who interviews the parent or caregiver and/or patient to document the criteria for the disorder. This comprehensive diagnostic process ensures that ADHD is correctly identified, distinguishing it from other disorders with similar symptoms, particularly externalising behaviour problems (Lafavor et al. 2022), reactions to stress, trauma, or substance use disorders. Homeless adolescents have a high prevalence of substance use disorder (Liu et al. 2022), which is consistent with the studies we reviewed. Substance use disorders and ADHD share common behavioural characteristics such as impulsivity, inattentiveness, and hyperactivity. It cannot be ruled out that the symptoms of substance use disorders can mimic those of ADHD. This overlap in symptomatology may lead to both underdiagnosis and overdiagnosis of ADHD within this population (Levin and Upadhyaya 2007).

Our results also suggest that homeless adolescents (mean age ≥ 12 years) are more likely to have ADHD than children (mean age < 12 years), whereas a meta-analysis not specifically dedicated to the homeless found a higher prevalence of ADHD in children aged 3–12 years (7.6%) compared with adolescents aged 12–18 years (5.6%) (Sayal et al. 2018). The social and family characteristics of homeless children differ from those of homeless adolescents. Indeed, in the studies we reviewed, with the exception of the Nepalese study (Ojha et al. 2013), children were systematically homeless with their families, whereas adolescents were homeless alone without their families. Homeless families are more likely to be homeless because of economic poverty, while homeless adolescents become homeless for a variety of reasons, including emotional, physical, or sexual abuse; behavioural problems; substance use disorder; or a psychiatric disorder in a parent (Shane 1996; Rew 2002; Liu et al. 2022). In addition, Cooper et al. observed that in a cohort of young adults, those who had been homeless and had at least one adverse childhood experience had a five-fold increased risk of ADHD (Cooper et al. 2023). Thus, having ADHD with comorbid behavioural or substance use disorders may favour homelessness, just as being homeless or experiencing other types of early adversity may favour the development or exacerbation of ADHD symptoms.

For homeless children and adolescents, a comprehensive approach to care must be prioritised. This includes addressing global health, social needs such as housing (e.g., through a Housing First approach) (Altena et al. 2010; Collins et al. 2019; Wang et al. 2019), and reintegration into education systems. Establishing stability in these areas is essential before conducting an evaluation for ADHD. However, given the high prevalence of ADHD in this population and the availability of effective treatments (Pouchon et al. 2023; Faraone et al. 2024) diagnosing and managing ADHD becomes a critical next step. Evidence-based therapies not only reduce symptoms and functional impairments but also improve long-term outcomes (Li et al. 2024). For instance, prolonged use of methylphenidate has shown protective effects against depression and substance use disorders without increasing risks for suicide or psychotic disorders (Inglis et al. 2016; Man et al. 2023). Additionally, delays in initiating treatment after diagnosis have been linked to higher mortality rates (Li et al. 2024). Despite these benefits, homeless children and their families often have limited contact with healthcare services (Macleod et al. 2023), while primary care providers may lack familiarity with their specific needs (Woan et al. 2013).

This study has several limitations that should be considered in future research. First, there was a high heterogeneity among the included studies due to differences in definitions, demographics, duration of homelessness, various measurement tools, and lack of comparison groups. We addressed the issue of heterogeneity using random-effects meta-analysis models as well as meta-regression and subgroup analyses. Gender was not always reported, and our meta-analysis found no difference in the prevalence of ADHD between the sexes using the F/M proportion, although previous studies indicated that boys are 2–3 times more likely to be diagnosed with ADHD than girls (Rucklidge 2010). Other co-morbidities (race, ethnicity, substance use) either not reported or poorly documented. Second, national homelessness policies regarding accommodation, prevention, coordinated approach, access to health services, and associated costs could influence mental disorders in homeless children and adolescents, particularly those with ADHD. Third, few studies have been conducted in low-income countries, although gender, culture, and the environment of homeless individuals (Scattolin et al. 2022) may affect the prevalence of ADHD (Al-Wardat et al. 2024). Fourth, we were unable to analyse the different situations of homelessness, particularly between individuals living on the streets often referred to as street homeless, who are challenging to reach—and those residing in shelters. Fifth, the studies included in this review were only cross-sectional in design. Longitudinal research is required to examine the trajectory of ADHD over time. They could help explain the difference in ADHD prevalence between children and adolescents that we observed. Future research should aim for consistency in methodology, target populations, and diagnostic criteria to improve comparability and reduce heterogeneity in ADHD prevalence studies.

## Conclusion

The review of 13 studies revealed that ADHD is common in homeless children and adolescents, suggesting that homelessness may contribute to the development or exacerbation of ADHD symptoms. Conversely, ADHD with other comorbidities may increase the likelihood of homelessness. Reintegrating these children and adolescents into care systems and ensuring access to public health interventions tailored for homeless families and youth is imperative for breaking the cycle of homelessness and improving long-term trajectories.

## Supplementary Material

Appendix .docx
