# Supplementary material for: Prevalence of attention deficit hyperactivity disorder in homeless children and adolescents: A systematic review and meta-analysis
Source: Dialogues Clin Neurosci. 2025 Apr 2;27(1):86–97. doi: 10.1080/19585969.2025.2486355 (PMC12312691; doi:10.1080/19585969.2025.2486355)
Supplement: Appendix .docx [file TDCN_A_2486355_SM0774.docx]

Appendix 1: Full search terms for database

**Pubmed:**

(("Attention Deficit Disorder with Hyperactivity"[MH] OR "Hyperkinesis"[MH] OR hyperactiv*[ALL] OR ADHD[ALL] OR "adhd's"[ALL] OR "AD/HD"[ALL] OR "ADH/D"[ALL] OR "Attention Deficit"[ALL] OR hyperkine*[ALL] OR ADHS[ALL] OR ADD[ALL] OR ADDH[ALL] OR inattent*[ALL]) AND ("Ill-Housed Persons"[MH] OR homeless*[ALL] OR "street people*"[ALL] OR "street liv*"[TI] OR vagrant*[ALL] OR destitute*[ALL] OR unhous*[ALL] OR "on the street*"[ALL] OR "rough sleep*"[ALL] OR "sleeping rough"[ALL] OR squatter*[ALL]))

**Scopus:**

(TITLE-ABS-KEY (( "Attention Deficit" OR "Hyperkine*" OR hyperactiv* OR adhd OR "adhd's" OR "AD/HD" OR "ADH/D" OR adhs OR addh OR adhds )) AND TITLE-ABS-KEY (( homeless* OR "street people*" OR "street liv*" OR vagrant* OR destitute* OR unhous* OR "on the street*" OR "rough sleep*" OR "sleeping rough" OR squatter* )))

**Psy Info:**

TI ((«Attention Deficit» OR «Hyperkine*» OR hyperactiv* OR ADHD OR «adhd's» OR «AD/HD» OR «ADH/D» OR ADDH OR ADHDs) AND (homeless* OR «street people*» OR «street liv*» OR vagrant* OR destitute* OR unhous* OR «on the street*» OR «rough sleep*» OR «sleeping rough» OR squatter*)) OR DE ((«Attention Deficit» OR «Hyperkine*» OR hyperactiv* OR ADHD OR «adhd's» OR «AD/HD» OR «ADH/D» OR ADDH OR ADHDs) AND (homeless* OR «street people*» OR «street liv*» OR vagrant* OR destitute* OR unhous* OR «on the street*» OR «rough sleep*» OR «sleeping rough» OR squatter*)) OR KW ((«Attention Deficit» OR «Hyperkine*» OR hyperactiv* OR ADHD OR «adhd's» OR «AD/HD» OR «ADH/D» OR ADDH OR ADHDs) AND (homeless* OR «street people*» OR «street liv*» OR vagrant* OR destitute* OR unhous* OR «on the street*» OR «rough sleep*» OR «sleeping rough» OR squatter*)) OR MJ ((«Attention Deficit» OR «Hyperkine*» OR hyperactiv* OR ADHD OR «adhd's» OR «AD/HD» OR «ADH/D» OR ADDH OR ADHDs) AND (homeless* OR «street people*» OR «street liv*» OR vagrant* OR destitute* OR unhous* OR «on the street*» OR «rough sleep*» OR «sleeping rough» OR squatter*)) OR MA ((«Attention Deficit» OR «Hyperkine*» OR hyperactiv* OR ADHD OR «adhd's» OR «AD/HD» OR «ADH/D» OR ADDH OR ADHDs) AND (homeless* OR «street people*» OR «street liv*» OR vagrant* OR destitute* OR unhous* OR «on the street*» OR «rough sleep*» OR «sleeping rough» OR squatter*)) OR SU ((«Attention Deficit» OR «Hyperkine*» OR hyperactiv* OR ADHD OR «adhd's» OR «AD/HD» OR «ADH/D» OR ADDH OR ADHDs) AND (homeless* OR «street people*» OR «street liv*» OR vagrant* OR destitute* OR unhous* OR «on the street*» OR «rough sleep*» OR «sleeping rough» OR squatter*)) OR AB ((«Attention Deficit» OR «Hyperkine*» OR hyperactiv* OR ADHD OR «adhd's» OR «AD/HD» OR «ADH/D» OR ADDH OR ADHDs) AND (homeless* OR «street people*» OR «street liv*» OR vagrant* OR destitute* OR unhous* OR «on the street*» OR «rough sleep*» OR «sleeping rough» OR squatter*))

**Web of Science**

((TS=(("Attention Deficit" OR "Hyperkine*" OR hyperactiv* OR ADHD OR "adhd's" OR "AD/HD" OR "ADH/D" OR ADHS OR ADDH OR ADHDs) AND (homeless* OR "street people*" OR "street liv*" OR vagrant* OR destitute* OR unhous* OR "on the street*" OR "rough sleep*" OR "sleeping rough" OR squatter*))) OR (TI=(("Attention Deficit" OR "Hyperkine*" OR hyperactiv* OR ADHD OR "adhd's" OR "AD/HD" OR "ADH/D" OR ADHS OR ADDH OR ADHDs) AND (homeless* OR "street people*" OR "street liv*" OR vagrant* OR destitute* OR unhous* OR "on the street*" OR "rough sleep*" OR "sleeping rough" OR squatter*))) OR (AK=(("Attention Deficit" OR "Hyperkine*" OR hyperactiv* OR ADHD OR "adhd's" OR "AD/HD" OR "ADH/D" OR ADHS OR ADDH OR ADHDs) AND (homeless* OR "street people*" OR "street liv*" OR vagrant* OR destitute* OR unhous* OR "on the street*" OR "rough sleep*" OR "sleeping rough" OR squatter*))) OR (KP=(("Attention Deficit" OR "Hyperkine*" OR hyperactiv* OR ADHD OR "adhd's" OR "AD/HD" OR "ADH/D" OR ADHS OR ADDH OR ADHDs) AND (homeless* OR "street people*" OR "street liv*" OR vagrant* OR destitute* OR unhous* OR "on the street*" OR "rough sleep*" OR "sleeping rough" OR squatter*))) OR (SU=(("Attention Deficit" OR "Hyperkine*" OR hyperactiv* OR ADHD OR adhd's OR "AD/HD" OR "ADH/D" OR ADHS OR ADDH OR ADHDs) AND (homeless* OR "street people*" OR "street liv*" OR vagrant* OR destitute* OR unhous* OR "on the street*" OR "rough sleep*" OR "sleeping rough" OR squatter*))) OR (WC=(("Attention Deficit" OR "Hyperkine*" OR hyperactiv* OR ADHD OR "adhd's" OR "AD/HD" OR "ADH/D" OR ADHS OR ADDH OR ADHDs) AND (homeless* OR "street people*" OR "street liv*" OR vagrant* OR destitute* OR unhous* OR "on the street*" OR "rough sleep*" OR "sleeping rough" OR squatter*))) OR (AB=(("Attention Deficit" OR "Hyperkine*" OR hyperactiv* OR ADHD OR "adhd's" OR "AD/HD" OR "ADH/D" OR ADHS OR ADDH OR ADHDs) AND (homeless* OR "street people*" OR "street liv*" OR vagrant* OR destitute* OR unhous* OR "on the street*" OR "rough sleep*" OR "sleeping rough" OR

squatter*))))

List of 22 excluded full-texts sought for retrieval

1. Shepherd, E. A. (2021). Homelessness and ADHD: A Hidden Factor?. <https://aura.antioch.edu/etds/755>
2. van Wormer, R. Homeless Youth Seeking Assistance: A Research-Based Study from Duluth, Minnesota. *Child & Youth Care Forum* **32**, 89–103 (2003). <https://doi.org/10.1023/A:1022589002915>
3. Salavera C, Antoñanzas JL, Bustamante JC, et al. Comorbidity of attention deficit hyperactivity disorder with personality disorders in homeless people. *BMC Res Notes*. 2014;7:916. Published 2014 Dec 16.
4. Pierce SC, Grady B, Holtzen H. Daybreak in Dayton: Assessing characteristics and outcomes of previously homeless youth living in transitional housing. Children and Youth Services Review, 2018;88,249-256
5. Trivedi C, Adnan M, Shah K, Manikkara G, Mansuri Z, Jain S. Psychiatric Disorders in Hospitalized Homeless Individuals: A Nationwide Study. *Prim Care Companion CNS Disord*. 2022;24(6):21m03209. Published 2022 Dec 15.
6. Busen NH, Engebretson JC. Facilitating risk reduction among homeless and street-involved youth. *J Am Acad Nurse Pract*. 2008;20(11):567-575.
7. Thompson RG, Hasin D. Psychiatric disorders and treatment among newly homeless young adults with histories of foster care. *Psychiatr Serv*. 2012;63(9):906-912.
8. Patterson ML, Moniruzzaman A, Frankish CJ, Somers JM. Missed opportunities: childhood learning disabilities as early indicators of risk among homeless adults with mental illness in Vancouver, British Columbia. *BMJ Open*. 2012;2(6):e001586. Published 2012 Nov 22.
9. Lomas B, Gartside PS. Attention-deficit hyperactivity disorder among homeless veterans. *Psychiatr Serv*. 1997;48(10):1331-1333
10. Merscham C, Van Leeuwen JM, McGuire M. Mental health and substance abuse indicators among homeless youth in Denver, Colorado. *Child Welfare*. 2009;88(2):93-110.
11. Santa Maria D, Padhye N, Yang Y, Gallardo K, Businelle M. Predicting Sexual Behaviors Among Homeless Young Adults: Ecological Momentary Assessment Study [published correction appears in JMIR Public Health Surveill. 2018 May 07;4(2):e10806. doi: 10.2196/10806]. *JMIR Public Health Surveill*. 2018;4(2):e39.
12. Nishio A, Yamamoto M, Ueki H, et al. Prevalence of mental illness, intellectual disability, and developmental disability among homeless people in Nagoya, Japan: A case series study. *Psychiatry Clin Neurosci*. 2015;69(9):534-542.
13. Waclawik K, Jones AA, Barbic SP, et al. Cognitive Impairment in Marginally Housed Youth: Prevalence and Risk Factors. *Front Public Health*. 2019;7:270. Published 2019 Oct 8.
14. Burke CW, Firmin ES, Lanni S, Ducharme P, DiSalvo M, Wilens TE. Substance Use Disorders and Psychiatric Illness Among Transitional Age Youth Experiencing Homelessness. *JAACAP Open*. 2023;1(1):3-11.
15. Morton LG 2nd, Cunningham-Williams RM. The capacity to give informed consent in a homeless population with developmental disabilities. *Community Ment Health J*. 2009;45(5):341-348.
16. Hesse M, Thiesen H. The use of the ADHD self-rating scale (ASRS-6) in the homeless: psychometric properties, alcohol use, and self-nurse agreement. *J Addict Nurs*. 2013;24(2):108-115.
17. Gomez RL, Janowsky D, Zetin M, Huey L, Clopton PL. Adult psychiatric diagnosis and symptoms compatible with the hyperactive child syndrome: a retrospective study. *J Clin Psychiatry*. 1981;42(10):389-394.
18. Special considerations for the health supervision of children and youth in foster care. *Paediatr Child Health*. 2008;13(2):129-136.
19. Santa Maria D, Daundasekara SS, Hernandez DC, Zhang W, Narendorf SC. Sexual risk classes among youth experiencing homelessness: Relation to childhood adversities, current mental symptoms, substance use, and HIV testing. *PLoS One*. 2020;15(1):e0227331. Published 2020 Jan 3.
20. Palines PA, Rabbitt AL, Pan AY, Nugent ML, Ehrman WG. Comparing mental health disorders among sex trafficked children and three groups of youth at high-risk for trafficking: A dual retrospective cohort and scoping review. *Child Abuse Negl*. 2020;100:104196.
21. Van Rooy (2008). Homeless not Hopeless: The Frequency of Characteristics of Attention Deficit Disorder.
22. Narendorf SC, Cross MB, Santa Maria D, Swank PR, Bordnick PS. Relations between mental health diagnoses, mental health treatment, and substance use in homeless youth. *Drug Alcohol Depend*. 2017;175:1-8.

Appendix 2: Risk of bias assessment

| Study | **Q1** | **Q2** | **Q3** | **Q4** | **Q5** | **Q6** | **Q7** | **Q8** | **Q9** |  | **Score** |
| --- | --- | --- | --- | --- | --- | --- | --- | --- | --- | --- | --- |
| Labelle (2023), Canada | Yes | No | Yes | Yes | Yes | Yes | Unclear | Yes | Unclear | living in temporary shelters | 6 |
| Asante (2015), Ghana | Yes | Yes | Yes | Yes | Yes | Yes | Yes | Yes | Unclear | Street children, CBCL/6-18 | 8 |
| Unger (1997), USA | Yes | Yes | Yes | Yes | Yes | No | No | Yes | Yes | Street or shelter, sampling, échelle de symptomes | 7 |
| Taib (Irak), 2014 | Yes | Yes | Yes | Yes | Yes | Yes | Yes | Yes | Yes | Street children, response rate donné (93%), echelle MINI | 9 |
| Ojha, 2013 | Yes | No | Yes | Yes | Yes | Yes | Yes | Yes | Not reported | foster homes | 7 |
| Da Silva (2010), Brazil | Yes | No | Yes | No | Yes | Yes | Yes | Yes | No | Recrutement peu clair, diagnostic par psychiatre | 6 |
| Roze (2016),  France | Yes | No | Yes | No | Yes | No | No | Yes | Yes | Taux de participation | 5 |
| Cauce (2000), USA | Yes | Yes | Yes | Yes | Yes | Yes | Yes | Yes | Yes | Taux de participation, vraie échelle | 9 |
| Zemanek (2020), USA | Unclear | Unclear | Yes | No | Unclear | No | No | Yes | No |  | 2 |
| Lewis (2017), USA | Unclear | Unclear | Yes | No | No | No | No | Yes | No |  | 2 |
| Yu (2008), USA | Unclear | Yes | Yes | No | Yes | No | No | Yes | No |  | 3 |
| Hayes (2013 ), USA | Unclear | No | Yes | No | Unclear | Yes | Yes | Yes | Yes | Taux de participation, échelle SDQ, foyers | 5 |
| Grant (2007), USA | Unclear | Yes | Yes | Yes | Yes | No | No | Yes | No |  | 5 |

The JBI Checklist provides quality criteria for prevalence studies in nine distinct items. The table indicates which items have been fulfilled for each included study, respectively:

Q1: Was the sample frame appropriate to address the target population?
Q2: Were study participants recruited in an appropriate way?
Q3: Was the sample size adequate?
Q4: Were the study subjects and setting described in detail?
Q5: Was data analysis conducted with sufficient coverage of the identified sample?
Q6: Were valid methods used for the identification of the condition?
Q7: Was the condition measured in a standard, reliable way for all participants?
Q8: Was there appropriate statistical analysis?
Q9: Was the response rate adequate, and if not, was the low response rate managed appropriate
